# Anthropometric measurements of patellar ridge using computed tomography-based three-dimensional computer models

**DOI:** 10.1186/s13018-021-02587-z

**Published:** 2021-07-06

**Authors:** Xiaoliang Mei, Hao Ding, Jia Meng, Jianning Zhao

**Affiliations:** 1 Department of Orthopedics, Jinling Hospital, Jinling Clinical Medical College of Nanjing Medical University, Nanjing, 210002 Jiangsu People’s Republic of China; 2 Department of Orthopedics, Taizhou Clinical Medical School of Nanjing Medical University, Taizhou, 225300 Jiangsu People’s Republic of China

## Abstract

**Background:**

The objectives of this study were to investigate the anatomic morphology of patellar ridge using computed tomography-based three-dimensional (3D) computer models and to assess the center of the patellar ridge after virtual resections.

**Methods:**

We selected 80 patients, 40 males (age, 33.2±6.8 years) and 40 females (age, 30.6±7.2 years), who were slightly symptomatic with soft tissue injury of the knee joint. The right or left knees were scanned by computed tomography (CT). The CT data of 160 knees was used to construct 3D computer models by image analysis software (Mimics). Variables such as the angle between the patellar ridge and patellar long axis, the distance between the center of the patellar ridge and the center of patellar cut after virtual resections were measured. We detect differences between the sides and genders with the 3D computer models by Student’s t test. Simple linear regression and correlation test was used to correlate the patellar ridge center to the center of the patellar cut.

**Results:**

According to the available data, there were significant gender differences in the length and width of patellar cut after virtual resections even with strict control for the height and weight of the patients. The angle between the patellar ridge and the patellar long axis was 11.24° ± 3.62°. The angle in male patients was 10.17° ± 4.82°, and it was 12.28°± 3.78° in female patients. The morphological difference was statistically significant (P < 0.05). After using the subchondral method to virtually resect the patellae, with reference to the center of the patellar cut, the center of the patellar ridge lies superiorly and medially in 88.75%, inferiorly and medially in 8.75%, laterally and superiorly in 2.5%, and in no case laterally and inferiorly. The intra-observer reliability regarding the dimensional measurements was excellent in this study.

**Conclusions:**

Advances in 3D computer models had resulted in the availability of preoperative measurement and virtual planning. The anthropometric dimensions of this study could provide general information for guiding surgical management of the patella in total knee arthroplasty (TKA) and were useful in designing patellar implants.

**Clinical relevance:**

The placement of the patellar component during TKA differs from one patella to another. The anatomic morphology information of the patellar ridge is helpful for surgeons to perform patellar resurfacing in TKA.

## Introduction

Despite the excellent clinical success of TKA, there is no consensus in the available literature on the best management of the patella in TKA [1]. One of the main controversies in TKA is the patella, with or without resurfacing [2, 3]. Although there are various studies comparing patellar resurfacing and non-resurfacing in TKA, the remarkable superiority of one plan over the other has not been described [4]. Meanwhile, both plans have their respective benefits and risks that need to be assessed and balanced based on the surgeon’s experience, preference, and patient’s expectations. Complications in relation to the patellar resurfacing such as patellar instability, anterior knee pain, and patellar clunk syndrome are usually due to the anatomy of the patient knee, the surgical technique, or the location of the patellar prosthetic [2]. However, with the surgical techniques and implant designs modified, patellar resurfacing has obtained increasing favor with surgeons [5, 6].

In order to gain satisfactory results, several principles should be followed during the procedure of the patellar resurfacing, including that the patellar prosthesis should be implanted in the right position to replicate the original high point of the patella [7, 8]. Detailed understanding of the ecological characteristics of the patellar ridge is essential for a successful surgery. However, there was few anthropometric data of the patellar ridge in the literature relative to that of the femur and tibia [9–11]. Besides that, it is difficult to identify the bony landmarks for the shape of the patella. The majority of the published articles performed measurements on the diseased knees, which could not recreate pre-morbid morphotype [12, 13]. Wiberg’s classification was helpful in identifying not only the shape of the patella facet, but also the high variability of the position of the patella ridge [14]. Our hypothesis is based on the fact that the center of the patellar articular surface moves with the location of the ridge. Therefore, the optimal center of the prosthetic patella should be located in the same ridge plane to be as faithful to the joint anatomy as possible. From previous studies, however, it is unclear which correlations exist between the center of the patellar ridge and the patellar cut after resection in the knee joint. Performing accurate locations and resections still remains challenging.

Advances in 3D computer models on account of radiographic images could be helpful for the preoperative measurement and virtual planning [15]. The primary objective of this study was to measure the angle and the length of the patellar ridge and to identify the gender size differences of the patellar cut after virtual resection. The secondary aim of this study was to explore the relation between the center of the patellar ridge and the center of the patellar cut. We expected to achieve a further understanding of the patellar ridge to provide a basis for positioning the patellar button in TKA and improving prosthetic designs.

## Materials and methods

### Patients and CT images

Institutional review board approval was obtained for a retrospective review of patient records and imaging studies. Eighty patients (40 males, 40 females) who were diagnosed with acute soft tissue injury of the knee joint between July 2016 and March 2018 were reviewed. The inclusion criteria were as follows: (i) age ranges from 20 to 40 years, (ii) height ranges from 160 to 180 cm, and (iii) 18.5 kg/m^2^ < body mass index (BMI) < 32.0 kg/m^2^.The exclusion criteria were as follows: (i) previous surgery of knee joint, (ii) presented with patellar instability, (iii) patellar dysplasia, and (iv) advanced patellofemoral arthritis. Cognizant consent has been obtained from the participants prior to CT examinations. The patients were placed supine on the scanner with the knee fully extended and the patella facing towards the ceiling. Both knees of each participant were examined by a slice CT scanner (120 kVp, 200 mA, Siemens Somatom Sensation 16, Erlangen, Germany) with rotation time 1 s, 1.0-mm thickness, and 1.0-mm slice gap. Low-dose CT scan protocol was applied to minimize the radiation exposure, which has a radiation dose equivalent to long-leg radiographs. The data of images was stored in the Digital Imaging and Communications in Medicine (DICOM) format.

### 3D reconstruction and measurement

**Fig. 1:**
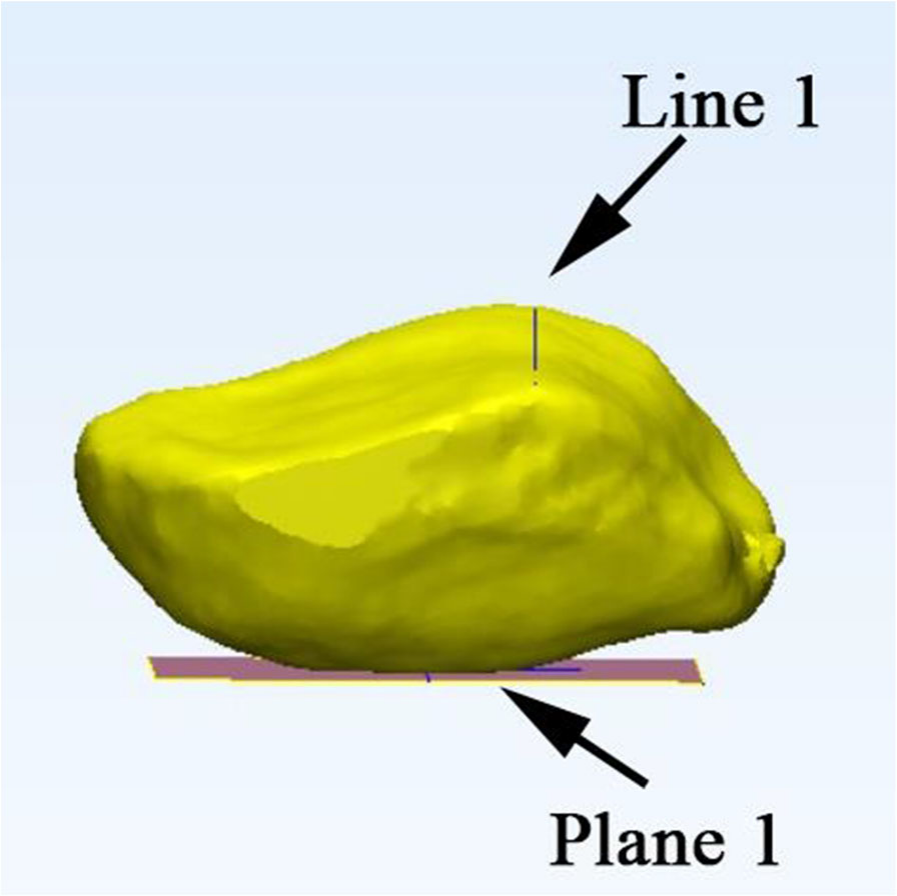
The three-dimensional model of the patella was established, and the patella was aligned with the fitted plane and line

We imported the CT scan images into the Mimics Software System, 17.0 (Materialise, Leuven, Belgium) and Geomagic Studio 2014 (Research Triangle Park, NC, USA) to establish 3D surface models. We developed a precise method to measure the patella in three dimensions. The Cartesian coordinate system of the CT data was converted into a new coordinate system. First, we selected multiple points on the anterior surface of the patella to fitting a base plane, which was performed automatically by a least-square method with the software. Subsequently, a line was connected similarly by selecting the two deepest points on the median ridge. Then, we adjusted the patella with its XY plane and Y-axis simultaneously parallel to the fitting base plane and line (Fig. 1). A new bounding box was created in this coordinate system automatically. Next, a virtual plane parallel to the fitting base plane was established. We translated the virtual plane along the Z-axis and set it at the deepest level of the lateral facet. The patella could be cut according to the virtual plane to simulate the subchondral resection method in routine TKA. After the model was established, various measurements were made in the new bounding box before and after the resection by an independent author who was blinded to the subjects identifying information (name, age, and sex). To assess the intra-observer reliability, the same author performed the measurements twice within an interval of 1 month.

### Parameter definitions

The stability of the patellofemoral joint is theoretically affected by the patellar ridge and by the position of the patella. We choose to measure several indexes to elaborate the characteristics of the patellar ridge, as follows:

#### The angle and location of the patellar ridge

**Fig. 2:**
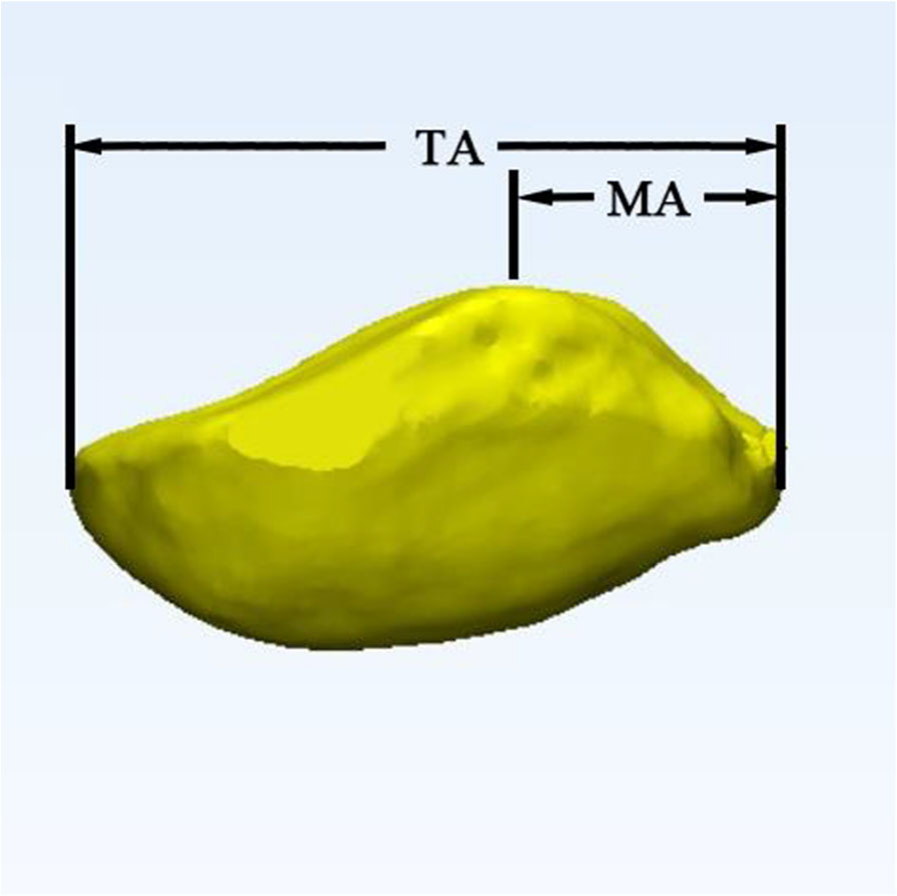
The ridge location was calculated by dividing the entire mediolateral width (TA) of the patella by the width of the medial facet (MA)

**Fig. 3:**
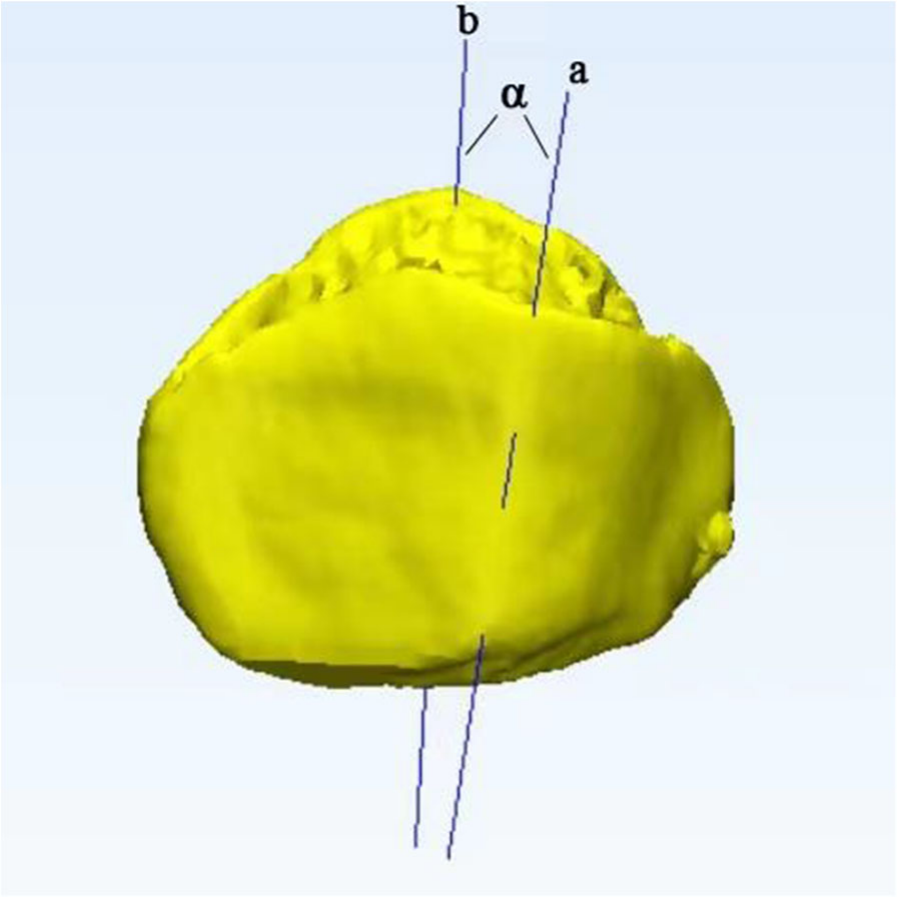
The angle (α) is between the patellar ridge (line a) and the long axis of the patella (line b), which is a line connecting the proximal and distal pole of the patella

The ridge location was calculated by dividing the whole mediolateral width of the patella by the width of the medial facet (Fig. 2). The angle of the patellar ridge represents the anatomic features of the patellar ridge, which is defined as the angle between the patellar ridge and the long axis of the patella (by selecting the proximal and distal poles of the patella and connecting them by a line) (Fig. 3).

#### The center of the patellar ridge

**Fig. 4:**
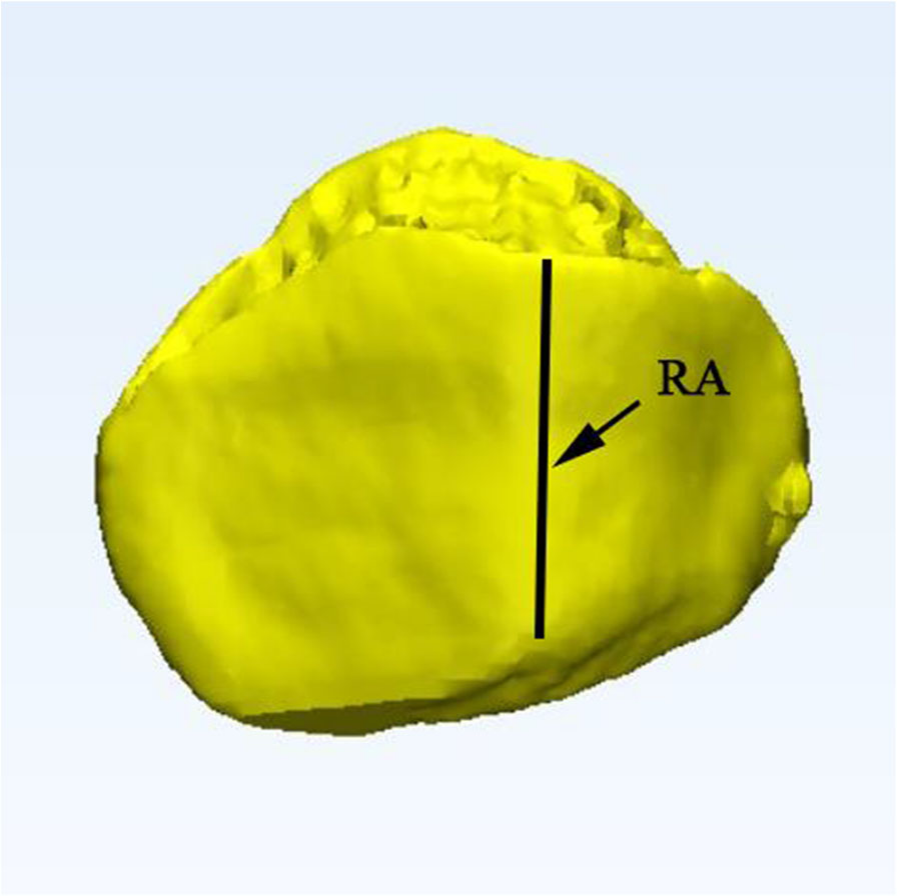
Measurement of the length of the patellar ridge (RA)

The patellar ridge is defined by positioning one point at the proximal end and one point at the distal end of the patellar ridge and then connecting them with the aid of a vertical line (Fig. 4). The midpoint of the vertical ridge was identified as the center of the patellar ridge which could be projected on the patellar cut.

#### The center of the patellar cut

**Fig. 5:**
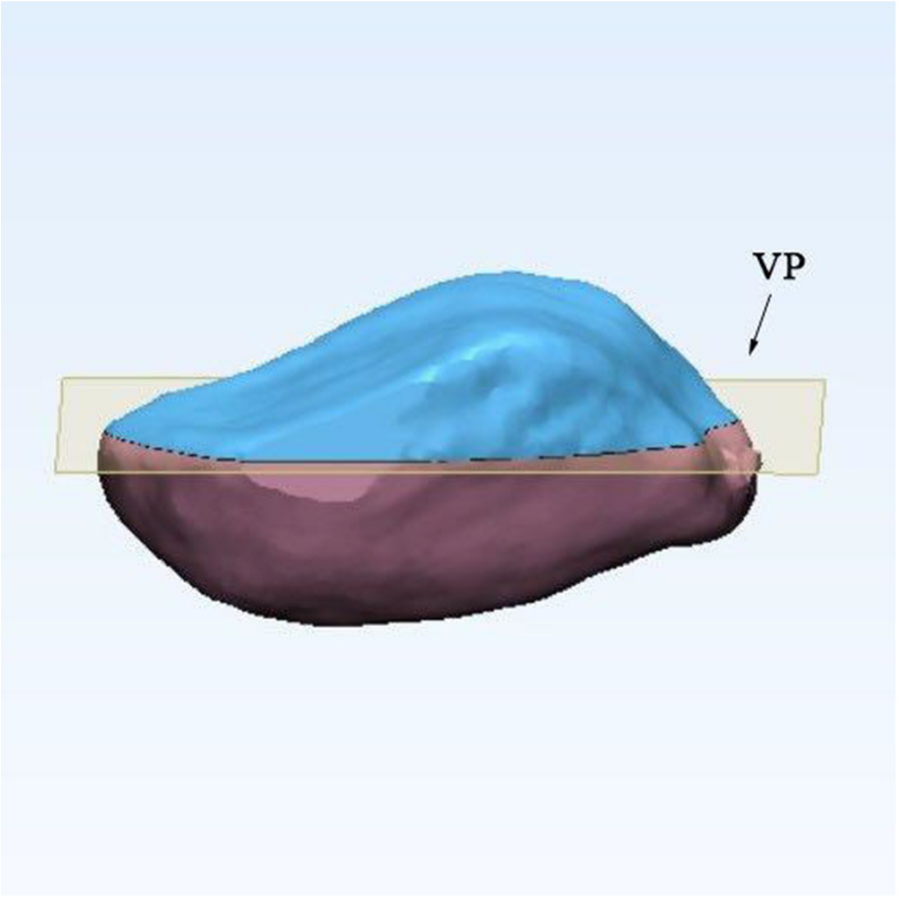
Creating a virtual plane (VP) parallel to the base plane, aligning the VP and setting it at the deepest level of the lateral facet

The patella was cut using the plane to simulate the subchondral resection method in routine TKA (Fig. 5). The center of the patellar cut was defined as the intersecting point of which over the vertical (proximal-distal) axis and the horizontal axis (medial-lateral).

#### Measuring method

##### Measurement of the angle between the patellar ridge and the long axis of the patella

After 3D reconstruction of the patella, we selected two points of the patellar ridge and connected them by a straight line, and the angle (α) is between the patellar ridge (line a) and the long axis of the patella (line b), which is a line connecting the proximal and distal pole of the patella.

##### Measurement of distance between the center of the patellar cut and the center of the patellar ridge

**Fig. 6:**
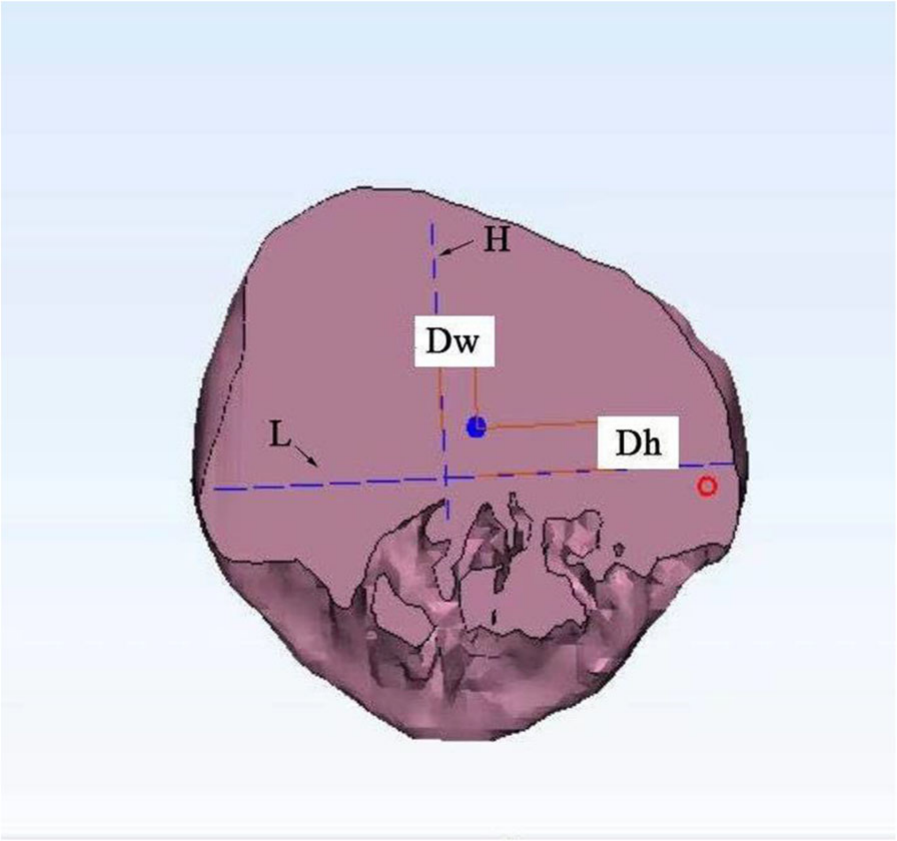
Measurement of the width (L) and the length (H) of the resecting surface, measurement of the values of patellar height and width along with the values of the horizontal distance (D_w_) and the vertical distance (D_h_) between the center of the patellar ridge and the center of the patellar cut

After the model was established, the patella was virtually cut by means of the plane to simulate the subchondral resection method in routine TKA. A vertical line (RA) was drawn along the patellar ridge and measured. The midpoint of the vertical ridge was projected onto the patellar cut. A cross was drawn on the patellar cut intersecting the horizontal and vertical axes where L and H represent the height and width of the patellar cut, respectively. The intersecting point of L and H was defined as the center of the patellar cut. The location of the patellar ridge center to the center of the patellar cut was then quantified via two measurements: one on the horizontal axis (medial-lateral distance) and another on the vertical axis (proximal-distal distance) (Fig. 6). The distances between the patellar ridge center and the center of the patellar cut were calculated in the horizontal plane [D_w_] and in the vertical plane [D_h_]. All the data was recorded in mm.

### Statistics

We used SPSS version 20.0 (IBM, Armonk, NY, USA) for statistical analysis. The intra-observer agreements were calculated by the intra-class correlation coefficient (ICC). The data was found to have a normal distribution which was tested by the Kolmogorov–Smirnov test. All the measured data are expressed as the mean and standard deviation. The differences in the variables between genders and the sides were analyzed with the t-test. In order to determine whether the size difference between genders is affected by morphological type, height and weight were used as covariates for covariance analysis. Simple linear regression and correlation test was used to correlate the patellar ridge center to the center of the patellar cut. Differences were considered to be significant at P < 0.05

## Results

The ICC values for the inter-rater reliability were greater than 0.81 for all measurements (ICC range from 0.89 to 0.93). Therefore, the measurement result reliability has been confirmed excellent. A total of 80 participants (40 males, 40 females) were enrolled in the study. The mean age of the male participants was 33.2 years (range, 20 to 40 years). The average height, weight, and body mass index (BMI) of the male group were 173.5 cm, 72.5 kg, and 24.8, respectively. Meanwhile, the mean age of the female participants was 30.6 years (range, 20 to 40 years). The average height, weight, and BMI of the female group were 162.4 cm, 60.7 kg, and 23.1, respectively. The demographics of the participants are generalized in Table 1.

**Table 1:** Clinical demographics of the male and female participants

	Male (n =40)	Female (n = 40)	P
Age (years)	33.2±6.8	30.6±7.2	0.12
Height (cm)	173.5±5.6	162.4±4.8	0.00
Weight (kg)	72.5±10.8	60.7±10.2	0.00
BMI (kg/m^2^)	24.8±3.4	23.1±2.8	0.00

### Comparison of the patellar ridge parameters between genders

The average patellar ridge length and the average angle between the long axis of the patella and the patellar ridge for men were 30.26 ± 2.33 mm and 10.17 ± 4.82°, respectively. The corresponding parameters for women were 28.16 ± 2.49 mm and 12.28± 3.78°, respectively. The mean (SD) values of the angle were 11.24°±3.62°. According to available data, there was a significant difference between the genders with the two parameters measured (Table 2). Meanwhile, there was no significant difference in the ridge position ratio between males and females.

**Table 2:** Comparison of the patellar ridge parameters between the genders

	Male (n = 40)	Female (n = 40)	P
Length of patellar ridge (mm)	30.26 ± 2.33	28.16 ± 2.49	0.00
Ridge position	0.42 ± 0.04	0.41 ± 0.05	0.32
Long axis angle of the patellar ridge and the patella (°)	10.17 ± 4.82	12.28± 3.78	0.03

### Comparison of the patellar ridge parameters between the sides

The statistical comparison results of measurement data of left and right knee joints were shown in Table 3. On the basis of the available data, no statistically significant differences were observed between these measurements (p > 0.05).

**Table 3:** Comparison of the patellar ridge parameters between the sides

	Left (n = 80)	Right(n = 80)	P
Length of patellar ridge (mm)	29.68 ± 2.68	29.73 ± 2.47	0.90
Ridge position	0.42 ± 0.03	0.41 ± 0.06	0.18
Long axis angle of the patellar ridge and the patella (°)	11.12± 4.12	11.08 ± 4.15	0.95

### Comparison of the patellar parameters between genders after the virtual resections

After virtual resection, the mean residual bony width and length were 44.36 ± 3.14 mm and 38.14 ± 3.86 mm, respectively, for men and 38.78 ± 3.05 mm and 34.52 ± 2.56 mm, respectively, for women. Basic measurements of residual bony width and length were significantly higher in men (P < 0.05). While no significance was found for medial displacement values between men and women (P > 0.05), the vertical distance between the center of the patellar ridge and the center of the patellar cut was significantly higher in men (P < 0.05). Mean (SD) values of patellar height and width along with the mean (SD) values of the horizontal distance and the vertical distance between the center of the patellar ridge and the center of the patellar cut were showed in Table 4.

**Table 4:** Comparison of the patellar parameters between genders after the virtual resections

	Male (n = 40)	Female (n =40)	P
Width of resecting surface (mm)	44.36 ± 3.14	38.78 ± 3.05	0.00
Length of resecting surface (mm)	38.14 ± 3.86	34.52 ± 2.56	0.00
Mean D_w_ horizontal distance	3.22 ± 1.65	2.68 ± 1.84	0.17
Mean D_h_ vertical distance	3.46± 2.32	1.82± 1.92	0.00

**Fig. 7:**
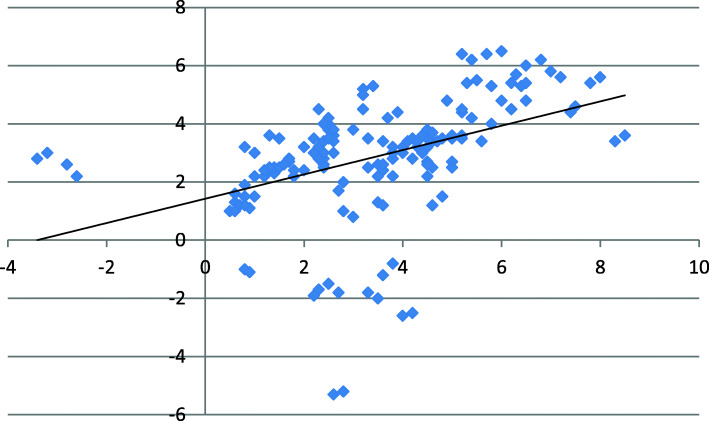
The points of the patellar ridge center were plotted on a scatter-plot graph with the reference point being the center of the patellar cut (0,0)

The points of the patellar ridge center were plotted on a scatter-plot graph with the reference point being the center of the patellar cut (0,0) (Fig. 7). In 97.5% (156/160) of the patellar models, the center of the patellar ridge was found to be medial to the center of the patellar cut while in 91.25% (146/160) of the cases, it was proximal to it. The correlation plot revealed the following: 88.75% (142/160) of the patients’ patellar ridge center was medial and superior, 8.75% (14/160) were medial and inferior, 2.5% (4/160) were lateral and superior, and 0% was lateral and inferior to the center of the patellar cut.

## Discussion

Our findings can be summarized as follows: (a) the patellar ridge was an oblique line from the proximal to the distal medial patella, which located in the medial patellar facet, and (b) the center of the patellar ridge could be located in three areas with regard to the center of the patellar cut: superiorly and medially in 88.75%, inferiorly and medially in 8.75%, and laterally and superiorly in 2.5%. The most important finding of this study was that the newly developed CT-based 3D computer model could be useful for measuring and virtually resecting the patella. The anthropometric measurements of the patellar ridge could provide basic data for guiding surgical management of the patella in TKA and are useful in designing patellar implants.

The function of the patella is to increase the lever arm of the extensor mechanism and make knee flexion more effective. It connects with the trochlear groove through its vertical ridge, dividing the patella into medial and lateral facet s[16]. Morphological analyses of the patella such as width and thickness of the patella have been conducted in previous studies; however, the patellar ridge has rarely been reported in the literature. Some authors have indicated that the patellar ridge can act as a fulcrum in the flexion activities of the knee joint, thus influencing normal kinematics [12]. Using cadavers, Gaurav et al. assessed the position of the patellar ridge from the proportion of the medial facet width within the whole width [17]. They found the patellar lateral and medial facet width ratio to be 1:3; the patella ridge was located in the medial facet. Yoo et al. reported that the central ridge of the patella was located 19.9 mm lateral from the medial border by means of magnetic resonance images [18]. Several scholars have investigated the anatomic characteristics of the patellar ridge and the matching relationship between the patellar ridge and the femoral trochlear groove. Their results indicate that the anatomic morphology of the patellar ridge allows the patella to engage with the trochlear groove. If the patellar ridge and femoral trochlear groove had a parallel relationship, that would impede the engagement of the patella into the trochlear groove [19]. Therefore, the patellar ridge is very important for the function of patellofemoral joint. Using CT and 3D reconstruction, we also found the patellar ridge to be an oblique line from the proximal to the distal medial patella, located in the medial patellar facet. This special structure of patellar ridge perhaps is of great significance for the diagnosis of patella instability and maltracking.

In TKA with the patella resurfacing, the poor position of the patellar prosthesis can lead to complications such as patellar instability, aseptic loosening, excessive wear, anterior knee pain, or implant failure [20]. How to get a proper position of the patellar prosthesis is an important technical issue in TKA, which has been less researched. Several technical factors may contribute to identify the optimal position of the patellar prosthesis. Medialization of the patellar prosthesis during patellar resurfacing has been recommended with less need for lateral retinacular release and a significant decrease in patellofemoral contact force [21]. Clinical studies have shown definite decreases in the rate of lateral retinacular release as a result of medialization of the patellar component [22]. With the consensus being in favor of medialization, many surgeons place the patellar component based on the position of the median ridge. Hofmann et al. emphasized that the surgeon should attempt to place the high point of the component at the location of the median ridge, which is the normal high point of the patella in the individual patient [23]. However, Lee et al. found that native patella vary widely in their axial plane alignments, and this was affected by the various possible positions of the median ridge. According to that data, a more medial placement of the median ridge tended to be associated with worse initial patellar tilt [24]. Increased lateral patellar tilt can cause several problems, including incorrect loading of both the patella and the extensor apparatus, patellar impingement on the prosthesis, and erosion [25]. On the other hand, in some patients such as East Asian patients who had small medial facet, replicating the original center of the patellar ridge the median ridge can lead to an overhang of the medial prosthesis. The native anatomy and kinematics of the patellofemoral joint vary by individual. From previous literature, it is unclear which correlations exist between the center of the patellar ridge and the patellar cut after resection in the knee joint. Although many authors advocate the intermediation of patellar components, and for all patients, their methods are usually empirical, such as no more than 2.5 mm or lack of quantification, such as the proposal to place buttons in two-thirds of the medial patella [26, 27]. Our study has some news findings; in 97.5% of the patients, the center of the patellar ridge was found to be medial to the center of the patellar cut. If the center of the patellar ridge was regarded as optimal position for patellar prosthesis, the results of this study were inconsistent with those found which reported an optimal position being medial to the center of the patellar cut in every case. Additionally, placement of the patellar component in relation to the vertical axis of the patellar cut has been even less reported. Lee et al. concluded that a distal placement of the patellar component should lead to decreased loading at higher knee flexion angles [28]. Our results show that the center of the patellar ridge was located more proximally relative to the center of the patellar cut in 91.25% of cases with the others located more distally. Of course, our data are based on a CT-based 3D computer model; further clinical confirmation is still needed. In accordance with these studies as well as our findings, we do not recommend providing a single solution for all patients; an individualized approach for an optimal location of the patellar prosthesis would be offered as an alternative.

Though the newly developed CT-based 3D computer model could be advantageous to evaluating patellar dimensions, there were also some limitations in this study. One limitation was that the CT scans used to construct the 3D patellae did not include the cartilage thickness, which should be taken into account when preparing for patella resection. The other limitation was that we measured the knees of 20- to 40-year-old healthy subjects only. The patella of an arthritic knee might be excavated and reduced substantially by degeneration. Finally, it is worth noting that a major limitation of the current study is that this was a purely anatomical study and had no clinical relevance. We do not know whether changes in the patella ridge have significant clinical consequences. Therefore, our interpretation of the data was based on the previously published reports, expert opinion, or basic scientific research, on what constitutes a good surgical technique for patella surface replacement. Despite these limitations, we believe that our study developed a valid and reproducible method for patellar measurement and virtual resection.

## Conclusion

In summary, our study has shown that advances in 3D computer models had resulted in the availability of preoperative measurement and virtual planning. The anthropometric dimensions provide basic data reflecting the normal anatomy of the patella. It is useful for performing patellar resurfacing in TKA and improving prosthetic designs.

## Data Availability

All the data and materials involving this article will be available upon request by sending an e-mail to the first author.

## Abbreviations

3D: Three-dimensional
CT: Computed tomography
TKA: Total knee arthroplasty
DICOM: Digital Imaging and Communications in Medicine
ICC: Intra-class correlation coefficient
BMI: Body mass index

## References

[1] Meneghini RM (2008). Should the patella be resurfaced in primary total knee arthroplasty? An evidence-based analysis. J Arthroplast.

[2] Johnson TC, Tatman PJ, Mehle S, Gioe TJ (2012). Revision surgery for patellofemoral problems: should we always resurface?. Clin Orthop Relat Res.

[3] He JY, Jiang LS, Dai LY (2011). Is patellar resurfacing superior than nonresurfacing in total knee arthroplasty? A meta-analysis of randomized trials. Knee.

[4] Tang XB, Wang J, Dong PL, Zhou R (2017). A meta-analysis of patellar replacement in total knee arthroplasty for patients with knee osteoarthritis. J Arthroplast.

[5] Pilling RWD, Moulder E, Allgar V, Messner J, Mohsen A (2012). Patellar resurfacing in primary total knee replacement a meta-analysis. JBJS.

[6] Barrack RL, Bertot AJ, Wolfe MW, Waldman DA, Milicic M, Myers L (2001). Patellar resurfacing in total knee arthroplasty. A prospective, randomized, double-blind study with five to seven years of follow-up. JBJS.

[7] Kim JH, Yoo BW, Kim CW (2015). Influence of the rotational alignment of the femoral and patellar components on patellar tilt in total knee arthroplasty. Knee surgery & related research.

[8] Keshmiri A, Springorum H, Baier C, Zeman F, Grifka J, Maderbacher G (2015). Is it possible to re-establish pre-operative patellar kinematics using a ligament-balanced technique in total knee arthroplasty? A cadaveric investigation. Int Orthop.

[9] Yifei D, Jeffrey E (2013). Comprehensive assessment of tibial plateau morphology in total knee arthroplasty: influence of shape and size on anthropometric variability. J Orthop Res.

[10] Mahfouz M, Fatah E, Bowers LS, Scuderi G. Three-dimensional morphology of the knee reveals ethnic differences. 2012;470:172–85. 10.1007/s11999-011-2089-2 PMC3237843 21948324

[11] Bo Y, Yu JK, Zheng ZZ, Lu ZH, Cheng JH (2012). Computed tomography morphometric study of gender differences in osteoarthritis proximal tibias. J Arthroplast.

[12] Kim TK, Chung BJ, Kang YG, Chong BC, Sang CS (2009). Clinical implications of anthropometric patellar dimensions for TKA in Asians. Clin Orthop Relat Res.

[13] Shang P, Zhang L, Hou Z, Bai X, Huang X (2014). Morphometric measurement of the patella on 3D model reconstructed from CT scan images for the southern Chinese population. Chin Med J.

[14] Tecklenburg K, Dejour D, Hoser C, Fink C (2006). Bony and cartilaginous anatomy of the patellofemoral joint. Sports Traumatology, Arthroscopy.

[15] Huang AB, Luo X, Song CH, Zhang JY, Yang YQ, Yu JK. Comprehensive assessment of patellar morphology using computed tomography-based three-dimensional computer models. Knee. 2015;22. 10.1016/j.knee.2015.05.010 26100317

[16] Winegar BA, Udayasankar UK (2012). The basic science of the patella: structure, composition, and function. The journal of knee surgery.

[17] Agnihotri G, Kaur R, Kalyan GS (2013). Patellar shape, nose pattem and facet configuration in 200 north. International Journal of Current Research and Review.

[18] Yoo JH, Yi SR (2007). The geometry of patella and patellar tendon measured on Knee MRI. Surg Radiol Anat.

[19] Wang XM, Liu HX, Niu JH, Duan GM, Wang F (2016). Relationship between the patellar ridge and the femoral trochlea in the patellar tracking. Orthop Surg.

[20] Lachiewicz PF, Soileau ES (2006). Patella maltracking in posterior-stabilized total knee arthroplasty. Clin Orthop Relat Res.

[21] D'Lima DD, Chen PC, Kester MA, Colwell CW (2003). Impact of patellofemoral design on patellofemoral forces and polyethylene stresses. JBJS.

[22] Lewonowski K, Dorr LD, Mcpherson EJ, Huber G, Wan Z (1997). Medialization of the patella in total knee arthroplasty. J Arthroplast.

[23] Hofmann AA, Tkach TK, Evanich CJ, Camargo MP, Zhang Y (1997). Patellar component medialization in total knee arthroplasty. J Arthroplast.

[24] Lee RH, Jeong HW, Lee JK, Choi CH (2017). Should the position of the patellar component replicate the vertical median ridge of the native patella?. Knee.

[25] Nikolaus OB, Larson DR, Hanssen AD, Trousdale RT, Sierra RJ (2014). Lateral patellar facet impingement after primary total knee arthroplasty: it does exist. J Arthroplast.

[26] Kawano T, Miura H, Nagamine R, Urabe K, Matsuda S, Mawatari T, Moro-Oka T, Iwamoto Y (2002). Factors affecting patellar tracking after total knee arthroplasty. J Arthroplast.

[27] Anglin C, Brimacombe JM, Wilson DR, Masri BA, Greidanus NV, Tonetti J, Hodgson AJ (2010). Biomechanical consequences of patellar component medialization in total knee arthroplasty. J Arthroplast.

[28] Lee TQ, Budoff JE, Glaser FE, Research R (1999). Patellar component positioning in total knee arthroplasty. Clin Orthop Relat Res.

